# Tourniquet-ALPPS is a promising treatment for very large hepatocellular carcinoma and intrahepatic cholangiocarcinoma

**DOI:** 10.18632/oncotarget.25538

**Published:** 2018-06-15

**Authors:** Victor López-López, Ricardo Robles-Campos, Robeto Brusadin, Asunción López-Conesa, Álvaro Navarro, Julio Arevalo-Perez, Pedro Jose Gil, Pascual Parrilla

**Affiliations:** ^1^ Virgen de la Arrixaca Clinic and University Hospital, IMIB, Murcia, Spain; ^2^ Department of Radiology, Memorial Sloan Kettering Cancer Center, New York, USA

**Keywords:** hepatocarcinoma, intrahepatic cholangiocarcinoma, ALPPS, two-stage hepatectomy

## Abstract

When very large hepatocellular carcinomas (HCCs) and intrahepatic cholangiocarcinoma (IHCCs) with insufficient future liver remnants are treated using associating liver partition and portal vein ligation for staged hepatectomy (ALPPS), the outcome is often poor. We therefore tested the efficacy of a modified version of that technique, tourniquet-ALPPS. A review of the literature examining outcomes of HCC and IHCC patients treated with ALPPS revealed the incidences of morbidity ≥ III and postoperative mortality to be respectively 20.7% and 16.1% among HCC patients and 50% and 45.4% among IHCC patients. In the present case series, in which HCC and IHCC patients were treated with tourniquet-ALPPS, median tumor size was 100 mm (range: 70–200 mm). After surgical stage I, there was no morbidity, no mortality and the median future liver remnant had increased at day 7 by 76%. In surgical stage II, 100% of tumors were resectable (8 right trisectionectomies, 5 with inferior vena cava resection). Two patients experienced serious morbidity ≥ IIIB and 1 patient died (11%). One- and 3-year overall survival was 75% and 60%, respectively. Thus tourniquet-ALPPS appears to be an effective alternative to classical ALPPS for the treatment of patients with HCC or IHCC.

## INTRODUCTION

Patients with very large hepatocellular carcinoma (HCC) or intrahepatic cholangiocarcinoma (IHCC) occupying the entire right hepatic lobe require extensive liver resection but are at risk of posthepatectomy liver failure (PHLF) because they have an insufficient future liver remnant (FLR) [1–12]. In these cases, portal vein embolization (PVE) [2, 13, 14] induces increases in volume of between 30% and 50% after 4–8 weeks, but increases the risk of drop-out by up to 30% [13, 15–19]. On the other hand, associating liver partition and portal vein ligation for staged hepatectomy (ALPPS) [20–24] induces hypertrophy faster than PVE (by more than 60% in only 7 days), but with higher morbidity and mortality [25–36]. Indeed, outcomes of ALPPS for malignant liver tumors are discouraging [26], with recent publications in the ALPPS Registry [36–39] reporting the poorest results for HCC, IHCC and perihilar cholangiocarcinoma (PHCC). There is thus no consensus regarding the indications for ALPPS in these cases.

We have developed an original variant of classical ALPPS, which we call “ALTPS” (associating liver tourniquet and portal vein ligation for staged hepatectomy) [40] or tourniquetALPPS [41]. This technique entails a lower surgical risk during the first stage since liver bipartition is not performed. In this report, we provide a systematic literature review of postoperative morbidity and mortality in HCC and IHCC patients treated with ALPPS and present postoperative results and oncological outcomes in cases of very large HCC (advanced stages of Barcelona Clinic Liver Cancer) (BCLC) [42] and IHCC involving the right lobe with a left insufficient FLR treated using tourniquet-ALPPS as a hypertrophy technique.

## RESULTS

### Systematic review of the literature

#### Hepatocellular carcinoma

Among a total of 101 HCC cases reviewed (Table 1) [29, 43–62], 11 patients were female and 90 were male. Median tumor size was 8.6 cm (range: 3.1–19.4 cm), and median age was 56 years (range 32–83). The short-term outcomes showed that among the 69 patients who were ultimately included in the bibliography review, 36 had at least one complication (52.1%); of those 21 patients (20.7%) had complications ≥ stage III. Ninety-day mortality (excluding patients from Serenari *et al.*, for whom 1-year mortality was determined) was 16.1% (15 patients). Data extracted from the case series [43–55] showed one patient who died postoperatively, and there were no data available for one other patient.

**Table 1:** Literature review in hepatocellular carcinoma: clinical cases, hospital series and world registry series

HCC CLINICAL CASES												
Author (reference)	Year	HCC	Tumor size (cm)	Ethiology	Gender	Age	Morbidity	≥IIIb morbidity	Type of complication	90-day mortality	Disease-free survival (months)	Overall Survival (months)
Sala *et al.* [43]	2012	1	-	–	F	77	No	No	–	No	No recurrence (15)	Alive (15)
Cavaness *et al.* [44]	2013	1	3.4	HCV	F	57	No	No	–	–	–	–
Brustia *et al.* [45]	2013	1	–	HBV	M	46	Yes	Yes	IIIB (Bile leak)	No	No recurrence (8)	Alive (8)
Chia *et al.* [46]	2014	1	16	HBV	M	55	No	No	–	No	No recurrence (2)	Alive (2)
Le Xiao *et al.* [47]	2015	1	6	Cirrhotic	–	–	No	No	–	No	No recurrence (4)	Alive (4)
Romic *et al.* [48]	2016	1	–	–	F	64	No	No	–	No	No recurrence (12)	Alive (12)
Cheung *et al.* [49]	2016	1	14	HBV	M	55	No	No	–	No	No recurrence (10)	Alive (10)
Torres *et al.* [50]	2016	1	19.4	Alcohol	M	57	No	No	–	No	No recurrence(3)	Alive (3)
Santibañes *et al.* [51]	2016	1	10	Cirrhotic	F	66	No	No	–	No	No recurrence (8)	Alive (8)
Papamichail *et al.* [52]	2016	1	8	Alcohol	M	68	Yes	No	I (Small for size)	No	Recurrence (6)	Dead (9)
Hong *et al.* [53]	2016	1	Multiple	HBV	M	43	Yes	No	II (Ascites)	No	–	–
Chen *et al.* [54]	2016	1	14	HBV	M	43	No	No	–	No	No recurrence (3)	Alive (3)
Sanei *et al.* [55]	2017	2	-	-	1F	48	Yes	Yes	V (PTE)	Yes	–	–
			-	-	1M	48	Yes	No	(PHLF and pleural effiusion)	No	No recurrence (34)	Alive (34)
**HCC SERIES**												
**Author**	**Year**	**HCC**	**Tumor size (cm)**	**Ethiology**	**Gender**	**Age**	**Morbidity**	**≥IIIb morbidity**	**Type of complication**	**90-day mortality**	**Disease-free survival (months)**	**Overall Survival (months)**
Álvarez *et al.* [56]	2015	3	-	-	-	-	-	33%	-	33	-	-
Vennarecci *et al.* [57]	2016	8	7.3 (3.1–14)	-	8M	65 (36–74)	100%	20%	-	12.5	-	-
Chan *et al.* [58]	2016	17	6 (2.5–15)	-	16M:1F	62 (50–80)	–	11.8%	-	5.9	–	-
Björsson *et al.* [59]	2016	4	10.4 (7–14)	-	4M	74.5 (68–83)	100%	0%	-	0%	50% (8.3)	50% (17.3)
Serenari *et al.* [29]	2016	8	-	-	6M:2F	56 (36–74)	62.5%	12.5%	-	60% (1y)	75% (1y)	62.5%
Chan *et al.* [60]	2017	25	7.5 (2 -16)	-	23M:2F	62 (50–80)	–	16%	-	8%	-	-
Wang *et al.* [61]	2017	10	9.2 (6.4–15.4)	-	9M:1F	41 (33–60)	50%	20%	-	30%	57.1% (7)	66% (7)
Cai *et al.* [62]	2017	12	8 (2.6–16)		10M:2F	43 (32–79)	70%	58.3%	-	50%	33% (16)	66% (16)
**HCC ALPPS REGISTRY**												
**Author**	**Year**	**HCC**	**Tumor size (cm)**	**Ethiology**	**Gender**	**Age**	**Morbidity**	**≥IIIA morbidity**	**Type of complication**	**90-day mortality**	**Disease-free survival (months)**	**Overall Survival (months)**
Schadde *et al.* [37]	2014	17 (8%)	**-**	**-**	**-**	**-**	–	25%	**-**	12%	87% (at 1 year)	61% (at 1 year)
Schadde *et al.* [63]	2015	32 (10%)	**-**	**-**	**-**	**-**	–	44%	**-**	13%	–	–
D’Haese *et al.* [38]	2015	35 (15.5%)	**-**	**-**	**-**	**-**	62.9%	26.9%	**-**	31.4%	60% (at 18 m)	68.6% (at 18 m)

HCC: hepatocellular carcinoma; F: female; M: male; PHLF: postoperative hepatic liver failure; PTE: Pulmonary *thromboembolism.*

Regarding the long-term oncological outcomes of the remaining 12 patients, one was lost during follow-up, and 11 were alive after 2–34 months of follow-up. In the hospital series [29, 56–62] and the World Registry of ALPPS publications [37, 38, 63], Overall survival (OS) and disease-free survival (DFS) ranged from 50–68.6 % and 50–87 %, respectively, though follow-up was less than 18 months (Table 1).

#### Intrahepatic cholangiocarcinoma

Of 12 patients studied in a case series [18, 57, 64–68] and hospital series [59] (Table 2), 7 were female and 5 were male. Median tumor size was 13.5 cm (range: 7–18 cm), and the median age of the patients was 61.5 years (range: 46–72 years). Among the short-term outcomes of these patients, 11 (91.6%) had postoperative complications, 6 of which were considered severe (50%), and 5 patients died (41.6%). Long-term oncological outcomes published in the World Registry of ALPPS series [37, 63] reported the OS and DFS to be 73% and 31%, respectively, after 1-year of follow-up.

**Table 2:** Literature review for intrahepatic cholangiocarcinoma: clinical cases, hospital series and world registry series

IHCC CLINICAL CASES											
Author	Year	*N*	Tumor size (cm)	Gender	Age	Morbidity	≥IIIB morbidity	Type of complication	90-day mortality	Disease -free Survival (months)	Overall Survival (months)
Troja *et al.* [64]	2014	1	−	F	72	Yes	Yes	V (Death, hemorragic shock)	Yes	−	−
Oldhafer *et al.* [65]	2015	1	−	M	46	Yes	No	II. Ascites	Yes	Recurrence (1)	Dead (2.5)
Vicente *et al.* [66]	2015	1	−	–	62	Yes	Yes	IVA (Biliary leak)	No	−	−
Vennarecci *et al.* [57]	2016	2	15	1F	60	Yes	Yes	V (Death, sepsis)	Yes	−	−
			12	1M	54	Yes	Yes	V (Death, sepsis)	Yes	−	−
Ulmer *et al.* [18]	2016	2	−	1M	72	Yes	Yes	IIIA (Biliary leak)	No	−	−
			−	1F	72	Yes	Yes	V (Pneumonia, Sepsis)	Yes	−	−
Lai *et al.* [67]	2017	1	18	F	50	No	No		No	No recurrence (3)	Alive (3)
Pineda-Solís [68]	2017	1	−	F	44	Yes	No	IIIA (Pneumonia, sepsis)	No	−	−
**IHCC HOSPITAL SERIES**											
**Author**	**Year**	***N***	**Tumor size (cm)**	**Gender**	**Age**	**Morbidity**	**≥IIIB morbidity**	**Type of complication**	**90−day mortality**	**Disease −free Survival (months)**	**Overall Survival (months)**
Björsson *et al.* [46]	2016	3	10.4 (7–14)	1M:2F	67 (61–70)	100%	0%	−	−	0%	66% (18)
**IHCC ALPPS REGISTRY**											
**Author**	**Year**	***N***	**Tumor size (cm)**	**Gender**	**Age**	**Morbidity**	**≥IIIB morbidity**	**Type of complication**	**90−day mortality**	**Disease −free Survival (months)**	**Overall Survival (months)**
Schadde *et al.* [37]	2014	8 (4%)	−	−	−	−	43%	−	13%	31% (at 1 year)	73 % (at 1 year)
Schadde *et al.* [63]	2015	13 (4%)	−	−	−	−	38%	−	15%	−	−

IHCC: intrahepatic cholangiocarcinoma; F: female; M: male.

### Case series

#### Demographic data

The patients included 6 men and 3 women with a median age of 60 years (range: 45–72). Median tumor size was 100 mm (range: 70–200 mm) (Table 3). There was retrohepatic vena cava invasion (IVC) of more than 50% of its circumference in 5 patients (2 HCC and 3 IHCC). On patient with IHCC exhibited invasion of the right portal vein and the bile duct causing jaundice (total bilirubin: 12.3 mg/dl), necessitating insertion of a percutaneous preoperative biliary drain.

**Table 3:** Demographic, volumetric, tumor, surgical and postoperative data of hospital series

	Case 1	Case 2	Case 3	Caso 4	Case 5	Case 6	Case 7	Case 8	Case 9
**Type of tumor**	HCC	HCC	HCC	HCC	IHCC	IHCC	IHCC	IHCC	IHCC
**Age (years)**	45	71	68	52	60	58	60	72	57
**Gender**	Male	Male	Male	Male	Female	Female	Female	Male	Male
**Histology**	Grade 2/6 fibrosis	Grade 2/6 fibrosis	Cirrhosis B virus	Cirrhosis B virus	Normal	Cholestasis	Normal	Normal	Normal
**Tumor size (n° nodules)**	200 (1)	120 (1)	70 + 40^*^ (2)	160 (1)	130 (1)	100 (1)	120 (1)	70 (1)	87 (1)
**BMI**	27.4	25	33	26	24	29.7	32.4	32.5	24.6
**Charlson index**	2	8	10	11	2	11	11	10	5
**MELD**	9	8	7	9	7	7	8	7	6
**Neoadjuvant treatment**	TACE	TACE	TACE	TACE	No	No	No	No	No
**Preoperative FLR (%)**	24	29	33	25	29	11	13	25	25
**FLR Before Stage II (%)**	69	44	47	48	60	31	39	44	45
**IFLR (%)**	187.5	51.7	42.4	105	76	182	200	76	50
**Stage II surgical tech.**	RT + IVCR	RT+IVCR	RH	RT	RT + IVCR	RT + IVCR+ PV+ Roux-HY	RT + IVCR	RT	RT
**Morbidity stage II (Clavien-Dindo)**	No	Chylothorax (IIIA)	Abscess (IIIB)	Ascites (II)	Chylothorax (IIIA)	Sepsis.PHLF (V)	No	No	Abscess IIIA
**Transfusion stage II (ml)**	1500	1200	No	300	600	1200	900	No	No
**Follow-up (months)**	Alive (60)	Died (5)	Alive (54)	Alive (4)	Alive (60)	Died (1)	Died (25)	Alive (54)	Alive (8)

HCC: hepatocellular carcinoma; IHCC: intrahepatic cholangiocarcinoma; IVCR: inferior vena cava resection; RPV: right portal vein; RT: right Trisectionectomy; RH: right hepatectomy; BMI: body mass index; TACE: transarterial chemoembolization; FLR: future liver remnant; IFLR: increase of future liver remnant volume. PHLF: postoperative hepatic liver failure. ^*^Both nodules in the right lobe.

#### Short-term outcomes

Surgical stage 1 was performed without the Pringle maneuver. Median blood loss was 50 ml (range: 50–600 ml), and surgical time was 125 min (range: 90–150 min). There was no morbidity or mortality, and the hospital stay was 6 days (range: 4–8 days). Median preoperative FLR was 25% (range: 11–33 %). By postoperative day 7, FLR had increased to 45% (range: 31–69 %). The median increase in FLR was 76% (range: 50–187.5 %), which corresponds to a daily increase of 28.5 ml/day (range: 7.5–110.7 ml/day). The preoperative FLR/body weight ratio increased from 0.40 (range: 0.17–0.62) to 0.77 (range: 0.49–1.18).

Surgical stage 2 was carried out a median of 13 days (range: 10–15 days) after stage 1, and the resectability was 100%. No Pringle maneuver was needed in any case. In the 2 HCC patients with hepatitis B viral cirrhosis, a right hepatectomy was performed in one case and a right trisectionectomy in the other. In the remaining 7 patients, a right trisectionectomy was performed, associating caudate lobe resection and IVC resection in 5 patients (2 HCC and 3 IHCC) (Table 3). The median blood loss was 750 ml (range: 100–1500 ml), and 5 patients were transfused. The surgical time was 285 min (range: 150–360 min). The median hospital stay was 9 days (range: 5–40 days). Five patients had complications ≥ stage IIIA, and 2 were ≥ stage IIIB Table 3). Two patients presented with PHLF and fulfilled International Study Group of Liver Surgery (ISGLS) criteria grades A and C. One 58-year-old woman with IHCC died due to sepsis and PHLF. She developed acute cholangitis caused by *Pseudomonas aeruginosa* infection on postoperative day 20 due to stenosis of the Roux H-Y requiring reoperation.

#### Long-term oncological outcome

None of the 3 HCC patients received adjuvant treatment, whereas 3 IHCC patients received 6 cycles of gemcitabine plus cisplatin. With a median follow-up time of 46 months (range: 4–60 months), the 1-year and 3-year OS were 75% and 60%, respectively. During follow-up, there was a recurrence in the lung in an IHCC patient alive at 60 months and receiving chemotherapy. Two patients died during follow-up: one HCC patient with IVC resection died after 5 months due to urinary sepsis, one IHCC patient died after 25 months due to acute myocardial infarction. Three of the 4 HCC patients remain alive after 60, 54 and 4 months, respectively; while 3 of the 5 IHCC patients remain alive after 60, 54 and 8 months, respectively.

## DISCUSSION

Currently available evidence indicates the incidence of morbidity and mortality is high among HCC and IHCC patients treated using ALPPS [26, 27, 38, 49, 50, 52, 54, 57, 58, 69, 70]. In the first edition of the ALPPS World Registry [37], the 90-day mortality was reported to be 9%, but it was higher for HCC (12%), IHCC (13%), perihiliar cholangiocarcinoma (27%) and gallbladder carcinoma (33%) than in cases of colorectal liver metastasis (CRLM) (8%). For that reason, subsequent editions of the ALPPS World Registry attempted to identify factors contributing to a poor prognosis, either preoperatively or after the first surgical stage, in order to prevent futile second interventions [39, 63].

Treatment of early stage HCC is liver resection or transplantation, whereas for patients with BCLS stage B HCC, the recommended treatment is TACE or palliative treatment. However, recent reports suggest surgical treatment can achieve prolonged survival in advanced HCC patients [71, 42, 72–74], even patients with IVC invasion, as occurred in a patient in this series (60 months survival). These findings may justify an aggressive surgical approach and support the use of ALPPS in patients with BCLC stage B HCC. However, D’Haese *et al.* [38] reported higher 90-day mortality among HCC patients than among those with CRLM (31% vs. 7%). They concluded that the risk associated with ALPPS remains prohibitive for most HCC patients and that ALPPS should only be performed in a highly select group of HCC patients younger than 60 years and with a low fibrosis grade. Similar results were obtained by Vennarecci *et al.* [57], who reported a postoperative mortality rate of 23.1%. In our literature review, we also found mortality to be high among both HCC (16.1%) and IHCC (45.4%) patients treated with ALPPS.

Several alternatives to the classical technique have been developed in an effort to reduce ALPPS-related morbidity [30, 40, 51, 53, 61, 75–78], especially to reduce the aggressiveness of stage 1. We started using tourniquet-ALPPS in our Department in 2011. With this technique, stage 1 does not include bipartition of the liver so as to minimize blood loss and substantially shorten the surgical time. In the present series, despite the large size of their tumors, no patient experienced any complications after stage 1, and all achieved sufficient hypertrophy after 7 days to perform stage 2, with no tumor progression. To avoid tumor growth after surgical stage 1, 4 HCC patients we administered 2 sessions of preoperative transarterial chemoembolization (TACE). The surgery in stages 1 and 2 was performed without using the Pringle maneuver, which is used in 24% of patients treated with ALPPS [37]. This is because liver partition was not performed in surgical stage 1, and in stage 2 the bipartition was carried out on the ischemic line left by the tourniquet. During stage 2, 5 patients were transfused but this was mainly related to the extreme liver surgery performed with complete IVC resection.

ALPPS-related mortality is also decreasing (from 17% to 4% in 2015), especially at centers with experience in the technique. Independent factors associated with mortality include risk adjustment in patient selection (*P* < 0.001) and use of less invasive techniques in stage 1 surgery (*P* = 0.019) [77]. Although our small sample size is a limitation, it is noteworthy that there was no mortality among the HCC patients in the present series, despite performance of a more aggressive surgical technique. The only patient who died was diagnosed with IHCC with portal vein and IVC invasion, who was a high-risk patient due to preoperative jaundice and Roux H-Y anastomosis.

Theoretically, HCC has a lower regenerative capacity because it usually arises against a background of cirrhosis or fibrosis. However, an earlier study showed that ALPPS is technically feasible and safe in HCC patients with cirrhosis, in whom it induces significant volume increases [57, 79]. D’Haese *et al.* [38] reported that hypertrophy was lower in HCC patients than in CRLM patients (47 vs. 76 %; *p* < 0.002) and was negatively correlated with the degree of fibrosis. But surprisingly, Vennarecci *et al.* [57] found that hypertrophy at 7 days was greater in cirrhotic patients than in normal liver (71.7% vs 64.8%, respectively). In the 3 HCC patients treated with tourniquet-ALPPS, the increase was 187.5%, 51.7%, and 42.4%, respectively.

There is currently no general recommendation about adjuvant chemotherapy for IHCC [80, 81]. Some authors suggest adjuvant chemotherapy in cases with lymphovascular and perineural invasion or positive resection margins. In the present study, the decision in favor of adjuvant chemotherapy (gemcitabine/cisplatin) was taken by our interdisciplinary tumor board considering tumor staging (large tumors and IVC invasion).

D´Haese *et al.* [38] reported that after ALPPS, OS for HCC patients was significantly shorter than for CRLM patients, with a DFS of 8 months for CRLM and 12 months for HCC. In a study from Vennarecci *et al.* [57], who reported only a short median follow-up (15 months), the 1-year OS and DFS for HCC were 74% and 42%, respectively. The median DFS was only 9 months, and 3 of 8 HCC patients experienced tumoral recurrence. Schadde *et al.* [37] reported a 1-year OS of 61% for HCC. The results for IHCC were worse, with a 1-year DFS rates of 31% (with R0 resectability of 86%). In the present series with tourniquet-ALPPS, 1- and 3-year OS for IHCC were 75% and 60%, respectively. One patient experienced recurrence in the lung at 50 months, but is alive at 60 months and is currently receiving chemotherapy. In the literature review, with a follow-up of less than 18 months for both HCC and IHCC, DFS was 50% and 31%, respectively.

This study has several limitations in line with previously published articles. They are related primarily to its retrospective nature and small sample. In the present study, however, patients received more aggressive treatment due to the higher rate of IVC resection than in previously published studies.

In conclusion, despite the aforementioned limitations, tourniquet-ALPPS appears to be a feasible option for treatment of patients with HCC beyond BCLC classification and with IHCC frequently involving the IVC. Tourniquet-ALPPS induces sufficient hypertrophy after 7 days with no tumor progression, and it enables acceptable long-term outcomes. More studies with larger numbers of patients are needed to confirm these results.

## METHODS

### Systematic review

A systematic review of the English language literature was performed based on recommendations of the Preferred Reporting Items for Systematic Reviews and Meta-analyses (PRISMA) statement [84], the Strengthening the Reporting of Observational Studies in Epidemiology statement [82], and the Assessment of Multiple Systematic Reviews tool [83].

#### Information sources and database searching

**Figure 1:**
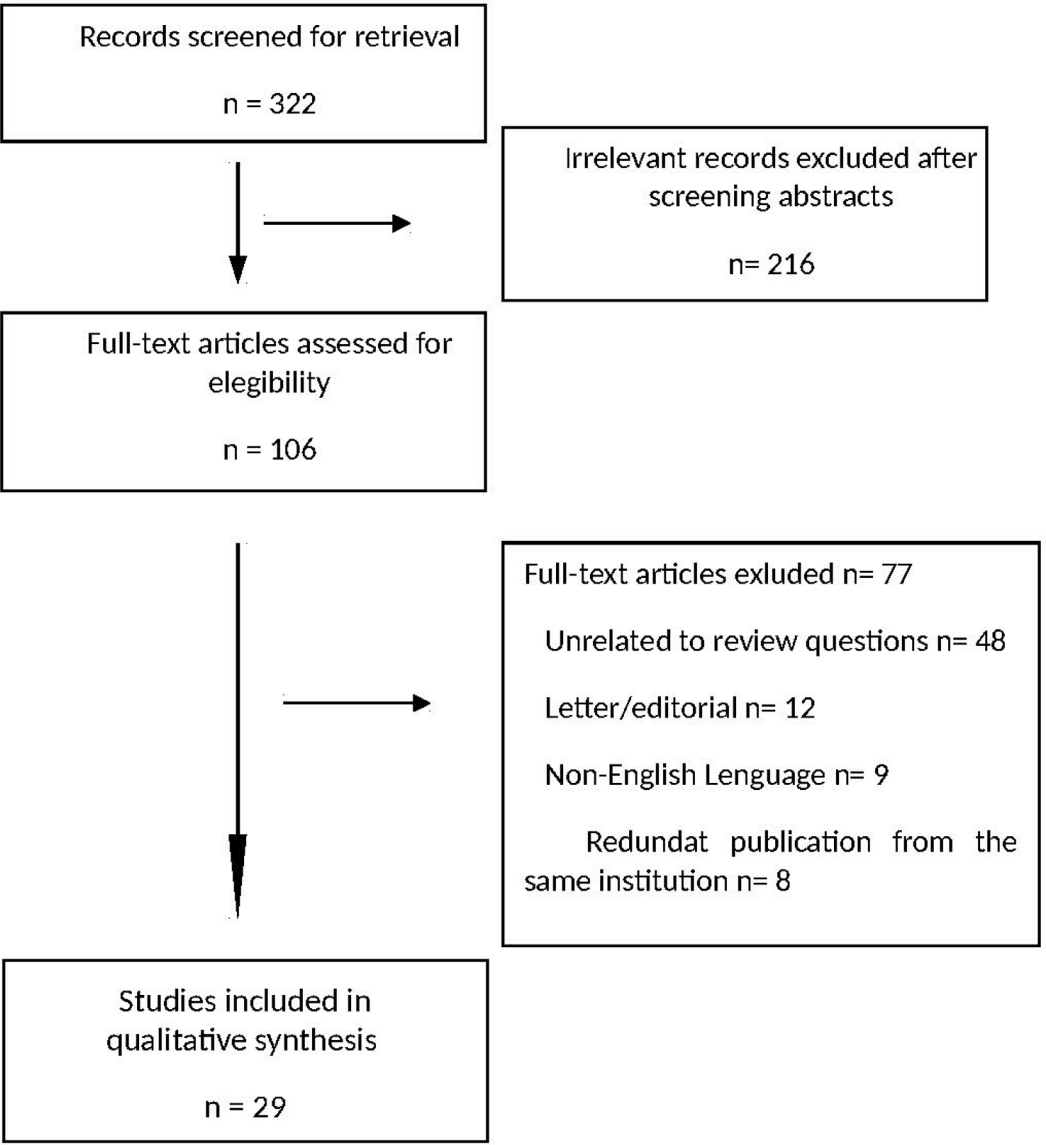
Modified PRISMA flow diagram of studies considered for inclusion in the systematic review

A medical librarian developed the systematic strategy utilized to search the Medline/PubMed, EMBASE, Scopus, ClinicalTrials.gov, the Cochrane Database of Systematic Reviews and the Cochrane Central Register of Controlled Trials. The search terms included a combination of standardized index terms and plain language to cover the terms “ALPPS”, “associating liver partition and portal vein ligation for staged hepatectomy” and “*in situ* split” as comprehensively as possible. Searches were limited to studies published in English using the standard limitations provided by the respective databases. Key review articles were identified, and their reference lists examined for relevant articles. The final search was performed in July 2017. Two researchers (VL & AN) independently screened bibliographies of relevant review articles and publications in the field. The same two researchers together screened titles and abstracts from the publications. In the event of disagreement, a third reviewer (RR) was involved. Refer to Figure 1 for a detailed flow schema, which was in accord with PRISMA guidelines.

#### Quality assessment

Study quality was assessed with the Cochrane risk of bias tool [84] using the following domains: sequence generation and allocation concealment; performance and detection bias; incomplete outcome data; selective outcome reporting; and other bias. Understandably, it is not feasible to conduct fully blinded studies for this research question, as both the patients and staff know the nature of the intervention. Given these difficulties, if a study did not mention any blinding of staff or patients and it was not possible to contact the authors, the study was assumed to be unblinded and therefore at high risk of performance and detection bias. It was, however, possible for detection bias to be reduced by using standardized criteria for complications and discharge and for outcome assessors to be unaware of the patients’ allocation.

#### Data extraction

Data were systematically extracted under the following headings: Study Design (e.g., Randomized Controlled trial, Registry review, Cohort study, etc), Study Population (Dates of recruitment, Number of patients, Age, Sex), Indications for HCC and IHCC, Surgical Procedures performed, morbidity, mortality and follow-up period.

#### Data synthesis

Data synthesis was performed using narrative methods. Because of the small number of studies assessed for methodological quality and the variety of outcome measures used, a meta-analysis was not possible.

### Case series

#### Patient selection

**Figure 2:**
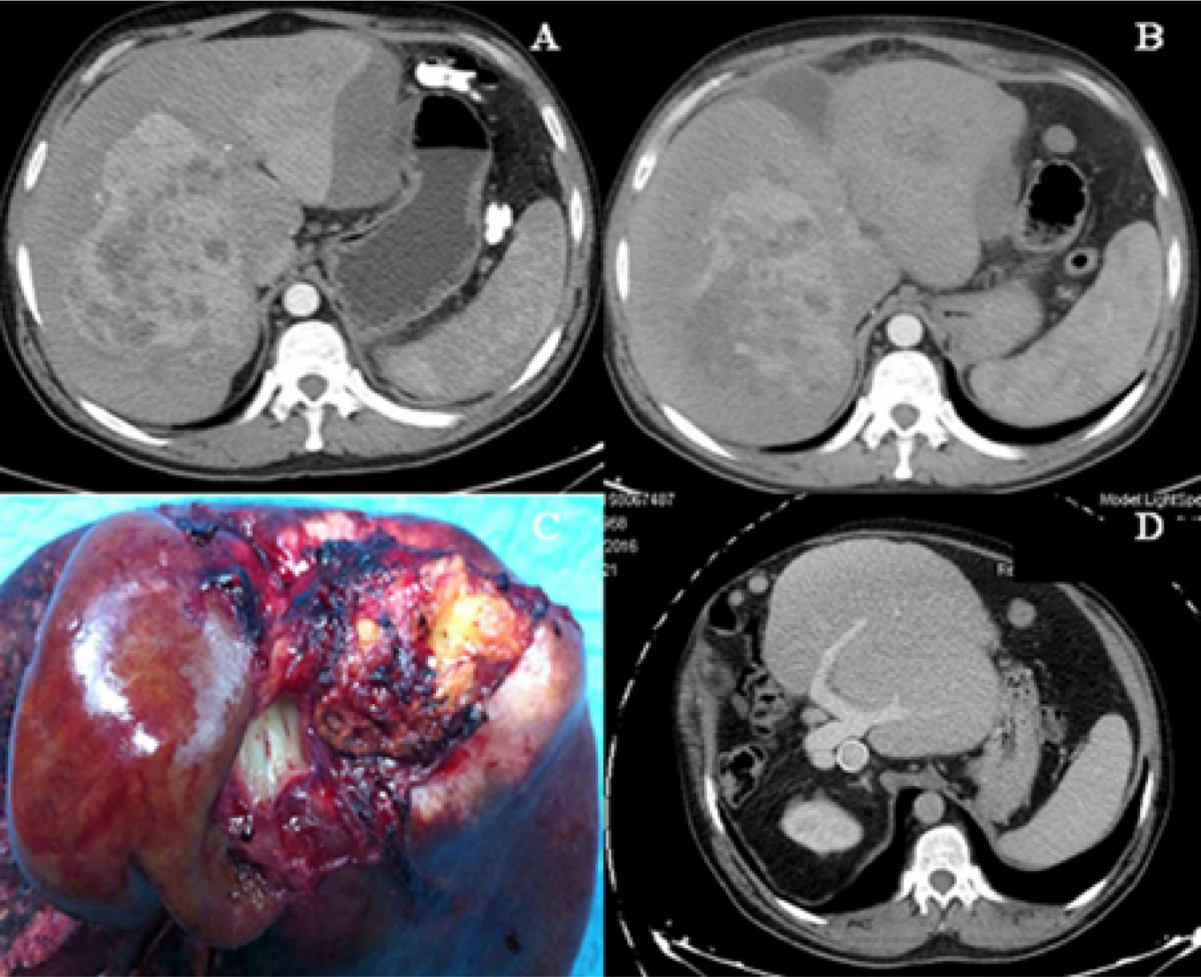
(**A**) Very large HCC with grade 2/6 fibrosis beyond the BCLC classification and inferior vena cava invasion; cirrhosis was ruled out, but subcapsular hematoma in the left lobe was detected. (**B**) CT volumetry on day 7 after stage I, with a future liver remnant increase of 187%. (**C**) Right trisectionectomy with inferior vena cava resection involving the caudate. (**D**) Follow-up CT at 60 months shows a disease-free liver with the vena cava graft still permeable.

**Figure 3:**
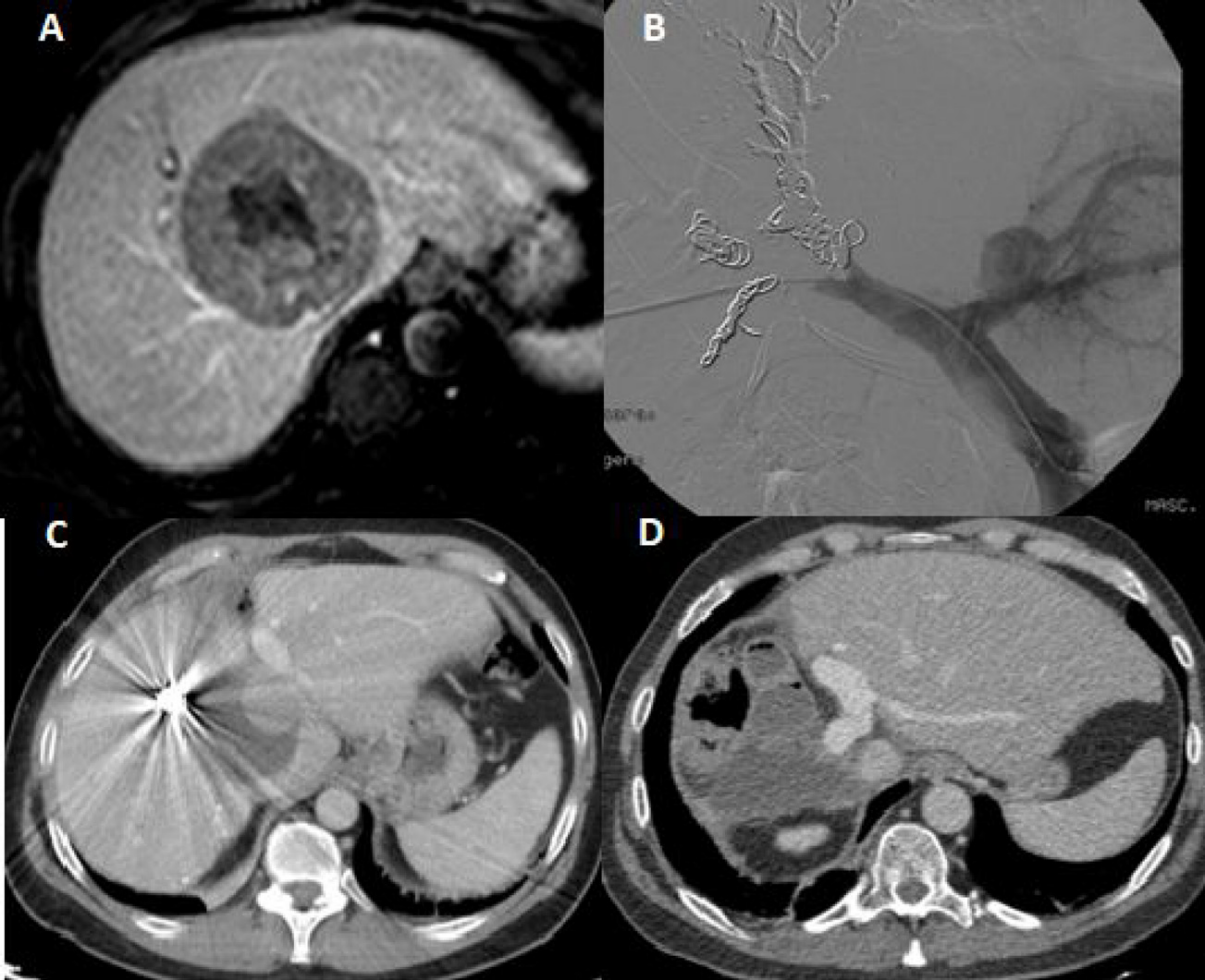
(**A**) Very large HCC with hepatitis B viral cirrhosis involving the right hepatic lobe. CT image depicting very large HCC involving the middle and right hepatic vein. (**B**) On day 4 after tourniquet placement during the first stage, we performed a right portal vein embolization. (**C**) CT volumetry on day 9 after stage I showing a future liver remnant increase of 105%. (**D**) Follow-up CT at 4 months shows the disease-free liver after right trisectionectomy.

Between September 2011 and July 2017, we performed one classical ALPPS and 50 tourniquet-ALPPS. Among the patients treated with tourniquet-ALPPS, 9 with HCC or IHCC were included in this study. Informed consent to be included in the study was obtained from all study participants. Tumor staging was carried out using the corresponding tumor markers (CEA, Ca 19.9, alpha-fetoprotein), CT (Figures 2 and 3), MRI and PET-scan. Exclusion criteria included the presence of extrahepatic disease and poor performance status (ECOG ≥ 2, ASA IV). Patients with HCC underwent TACE [85]. We assessed 90-day morbidity and mortality and long-term results. Morbidity was classified using Clavien-Dindo criteria [86], while PHLF was classified using ISGLS criteria [87].

Total liver volume (TLV), tumor volume, FLR (ml and percentage) and the FLR/body weight ratio were calculated. A FLR lower than 25% in patients with healthy livers or less than 50% in cirrhotic patients with intermediate stage or Child A cirrhosis were considered insufficient. A FLR/body weight ratio of less than 0.5 in patients with a healthy liver or less than 0.7 in those with a cirrhotic liver was considered insufficient.

#### Surgical technique

Tourniquet-ALPPS has been described previously [40]. In stage 1, after ligation of the right portal vein, we do not perform the liver partition. The tourniquet is placed within the umbilical fissure or main portal fissure and is tightened enough to occlude vascular communication between both lobes (which avoids liver transection but leads to the desired liver hypertrophy before resection is performed). The patients can usually be discharged a few days after the first stage. CT scan volumetry is performed on the 7th postoperative day (Figures 2 and 3) to assess the increase in FLR and FLR/body weight ratio.

In stage 2, we use the inserted tourniquet as an aid for the hanging maneuver, after which liver bipartition is performed using an anterior and *in situ* approach along the ischemic line caused by the tourniquet. In case of IVC invasion, after resection of the IVC, it was replaced with a 2-cm ringed Gore-Tex^®^ graft (Gore^®^, Newark, DE, USA) (Figure 2). Once the patients recovered from the surgery, those with IHCC received adjuvant chemotherapy in consultation with an oncologist. No patient with HCC received adjuvant chemotherapy.

### Statistical analysis

Statistical analysis was carried out using SPSS Statistics 22.0 software (SPSS Inc., Chicago, IL, USA). Kaplan-Meier curves were used for survival assessment.
